# Complementary Metamaterial Sensor for Nondestructive Evaluation of Dielectric Substrates

**DOI:** 10.3390/s19092100

**Published:** 2019-05-07

**Authors:** Tanveer ul Haq, Cunjun Ruan, Xingyun Zhang, Shahid Ullah

**Affiliations:** 1 School of Electronic and Information Engineering, Beihang University, Beijing 100191, China; tanveerulhaq@buaa.edu.cn (T.u.H.); luckyzhang@buaa.edu.cn (X.Z.); shahidkhan@buaa.edu.cn (S.U.); 2 Beijing Key Laboratory for Microwave Sensing and Security Applications, Beihang University, Beijing 100191, China

**Keywords:** complementary metamaterial sensor, CCSR, nondestructive evaluation, material under test, permittivity, transcendental equation

## Abstract

In this paper, complementary metamaterial sensor is designed for nondestructive evaluation of dielectric substrates. The design concept is based on electromagnetic stored energy in the complementary circular spiral resonator (CCSR), which is concentrated in small volume near the host substrate at resonance. This energy can be employed to detect various electromagnetic properties of materials under test (MUT). Effective electric permittivity and magnetic permeability of the proposed sensor is extracted from scattering parameters. Sensitivity analysis is performed by varying the permittivity of MUT. After sensitivity analysis, a sensor is fabricated using standard PCB fabrication technique, and resonance frequency of the sensor due to interaction with different MUT is measured using vector network analyzer (AV3672series). The transcendental equation is derived for the fabricated sensor to calculate relative permittivity for unknown MUTs. This method is very simple and requires calculating only the resonant frequency, which reduces the cost and computation time.

## 1. Introduction

Recently there has been increased growth in utilization of microwave sensors to improve quality assurance in various fields like food [1], healthcare [2], agriculture [3], and environment [4]. Advantages like low cost, ease of fabrication, robust design, and integration with other microwave devices are the main reasons for the popularity of microwave sensors based on complementary metamaterials (MTMs) [5]. Complementary MTMs are manufactured artificially and their properties depend on structure and orientation rather than composition. The first complementary MTM structure was introduced by F. Falcone et al. in 2004 [6] by applying duality argument on split ring resonator (SRR), which was introduced by Pendry et al. as a resonant magnetic particle in 1999 [7]. SRR consisted of two concentric copper rings having a small gap between them and split in each ring to support resonance. SRR acts as an *LC* resonator where the circumference of metallic rings provide inductance while the gap between the rings and splits provides the capacitance. The resonance frequency of SRR depends on the size of splits, diameter, and gap between the rings. Meanwhile, complementary split ring resonator (CSRR) is a negative image of SRR, which can be obtained by etching out SRR from a metallic plate. The intrinsic circuit and excitation model for isolated and coupled SRR and CSRR structures have already been discussed in Ref. [8].

Microwave sensors based on SRRs are usually magnetically coupled with the microstrip transmission line, therefore, these structures are etched on the top layer of microwave sensor near the microstrip line [9]. At the resonance frequency of SRRs, an electric field appears near the narrow split region, which can be employed for liquid characterization [10] and biomedical sensing [11]. SRRs-based sensors are not suitable for microwave sensing of large samples, because of the narrow fringing electric fields. This problem has been solved by utilizing CSRRs in place of SRRs for the measurement of dielectric samples with large dimensions [12]. CSRRs are usually electrically coupled with the microstrip transmission line and etched in the ground plane [13]. At resonance of CSRRs, large fringing electric fields appear in the ground plane, which can be employed to evaluate dielectric thickness [14] and loss tangent [15]. CSRRs have also been used for the synthesis of highly sensitive sensors for concentration of fluid materials [16], and identification of different liquids from a mixture [17].

In this paper, we are using a complementary circular spiral resonator (CCSR) to design a microwave sensor for nondestructive evaluation of dielectric substrates. Electric field concentration, effective electric permittivity, and magnetic permeability of the proposed sensor are calculated numerically. A sensor is fabricated and the magnitude of transmission coefficient (S_21_) is measured using vector network analyzer (AV3672 series). Five dielectric materials (MUTs) are placed on the fabricated sensor and S_21_ of the sensor is measured due to interaction with these MUTs. This measured data is used to derive a transcendental equation for the sensor. The sensor design is explained in Section 2. Sensitivity analysis is performed in Section 3. Fabrication and measurement results are discussed in Section 4. The transcendental equation is formulated in Section 5 and the research is concluded in Section 6. 

## 2. Sensor Design

**Figure 1:**
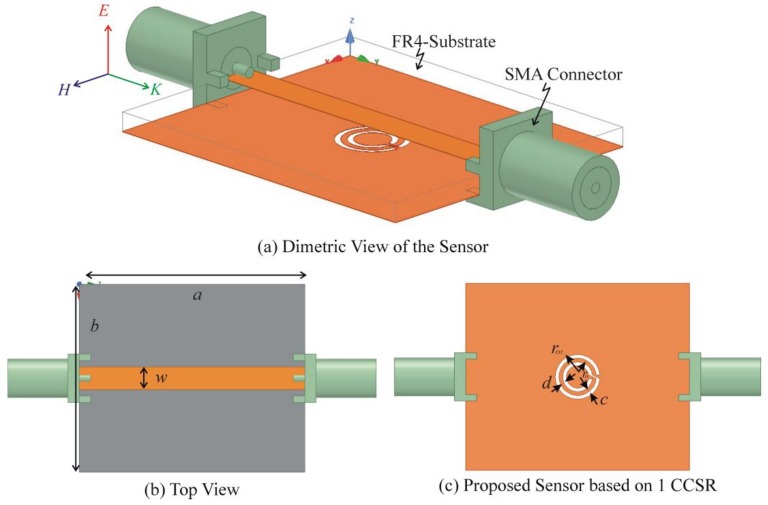
(**a**) Sensor design based on FR4 epoxy substrate (*h* = 1 mm). (**b**) Top view of the sensor (*a* = 30 mm, *b* = 25 mm, *w* = 3 mm). (**c**) Bottom view of the sensor (*r_ext_* = 3 mm, *r*_0_ = 1.5 mm, *c* = *d* = 0.5 mm).

The proposed sensor is based on 1 mm thick FR4 substrate, which has two copper layers of 35 µm each. The upper copper layer consists of a microstrip transmission line while the bottom copper layer has a complementary circular spiral resonator (CCSR) as shown in Figure 1. The dielectric constant (*ε_re_*) and characteristic impedance (*Z_c_*) of microstrip transmission line are calculated using the following equations [18]:

(1) $$\varepsilon_{re}=\frac{\varepsilon_{r}+1}{2}+\frac{\varepsilon_{r}-1}{2}{\left(1+12\frac{h}{w}\right)}^{-0.5}$$

(2) $$Z_{c}=\frac{\eta }{\sqrt{\varepsilon_{re}}}{\left\{\frac{w}{h}+1.393+0.677\ln\left(\frac{w}{h}+1.4444\right)\right\}}^{-1}$$

where *ε_r_* = 4.4 is permittivity of dielectric substrate and *η* = 120π Ω is impedance of wave in free space, *w* = 3 mm is width of the microstrip transmission line, *h* = 1 mm is height of FR4 epoxy substrate. The proposed sensor is simulated in ANSYS Electronics Desktop 2018, which includes a direct link to a high frequency structure simulator (HFSS). The proposed sensor is excited with electrical polarization in z direction, magnetic polarization in x direction, wave vector in y direction, and the simulation conditions are given in Table 1. 

**Figure 2:**
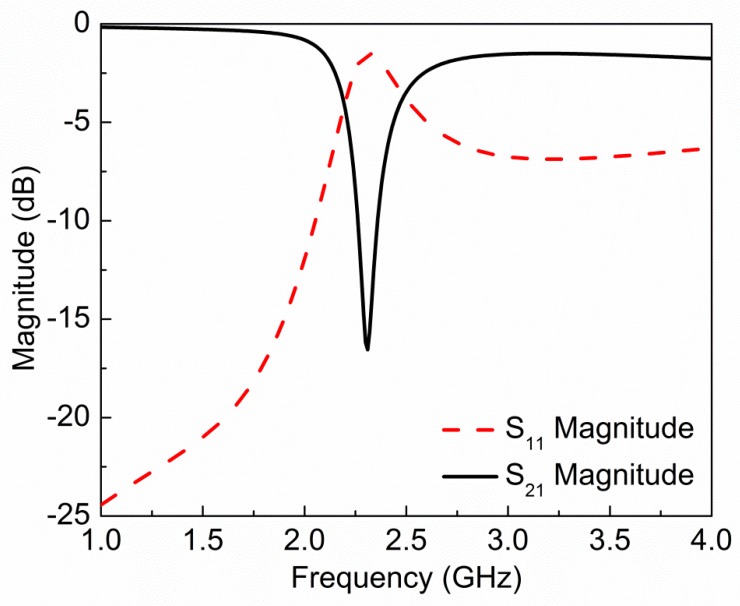
Magnitude of simulated reflection (*S*_11_) and transmission (*S*_21_) coefficient for the proposed sensor. Resonance frequency of sensor is 2.32 GHz with notch depth −16.54 dB.

**Figure 3:**
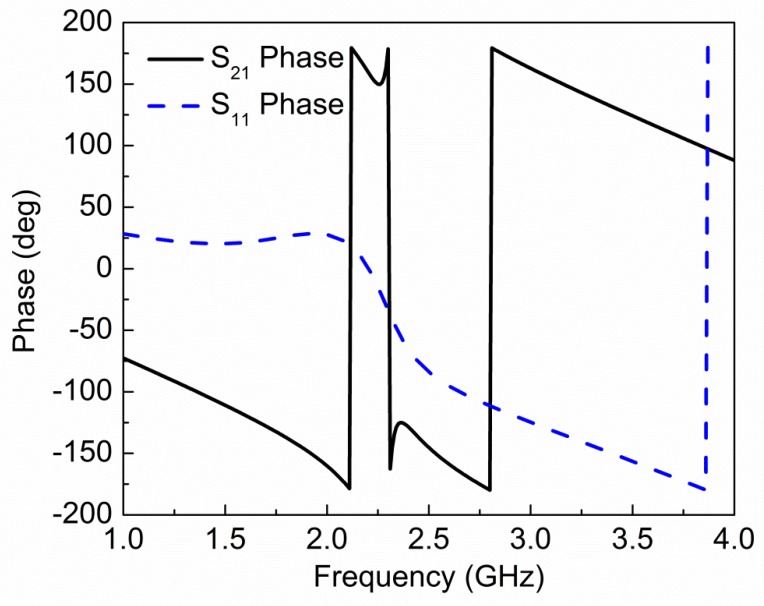
Phase of simulated reflection (*S*_11_) and transmission (*S*_21_) coefficient for the proposed sensor. A sudden change in phase occurs at resonance frequency.

The magnitude and phase of simulated transmission (*S*_21_) and reflection (*S*_11_) coefficients are shown in Figure 2; Figure 3, respectively. The fundamental resonance frequency of the sensor is 2.32 GHz with notch depth −16.54 dB. Scattering parameters are used to extract effective electric permittivity (*ε*) and magnetic permeability (*µ*) of the proposed sensor using the following relations [19]:

(3) $$\varepsilon =\frac{n}{z}$$

(4) μ=nz

where *n* is refractive index which can be calculated using following equation:

(5) n=1kt{Im[ln(einkh)]−iRe[ln(einkh)]}

where,

(6) $$e^{inkh}=\frac{S_{21}}{1-S_{11}R_{01}}$$

where,

(7) $$R_{01}=\frac{z-1}{z+1}$$

where *z* is the complex impedance which can be calculated using following equation:

(8) $$z=\pm \sqrt{\frac{{\left(1+S_{11}\right)}^{2}-S_{21}{}^{2}}{{\left(1-S_{11}\right)}^{2}-S_{21}{}^{2}}}$$

**Figure 4:**
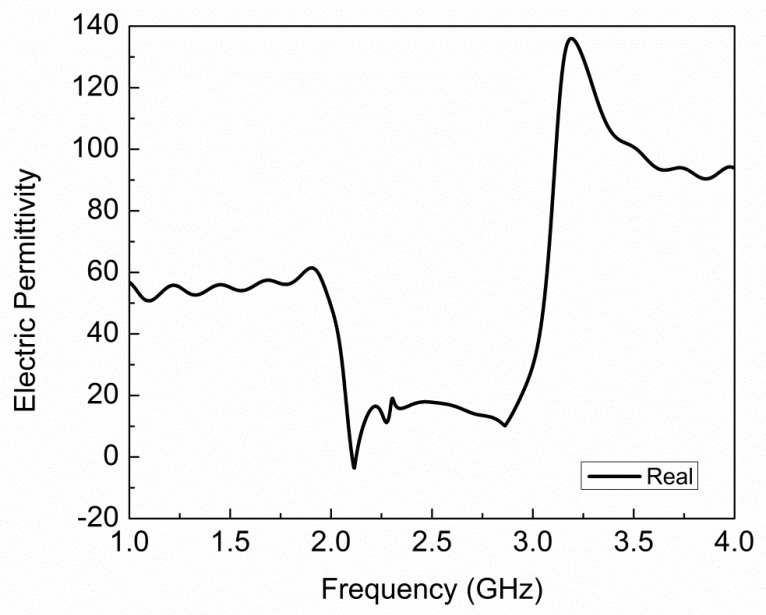
Extracted plot of electric permittivity for proposed sensor, showing negative permittivity from 2.10 GHz to 2.12 GHz, and maximum value of negative permittivity is −3.57 at 2.11 GHz.

**Figure 5:**
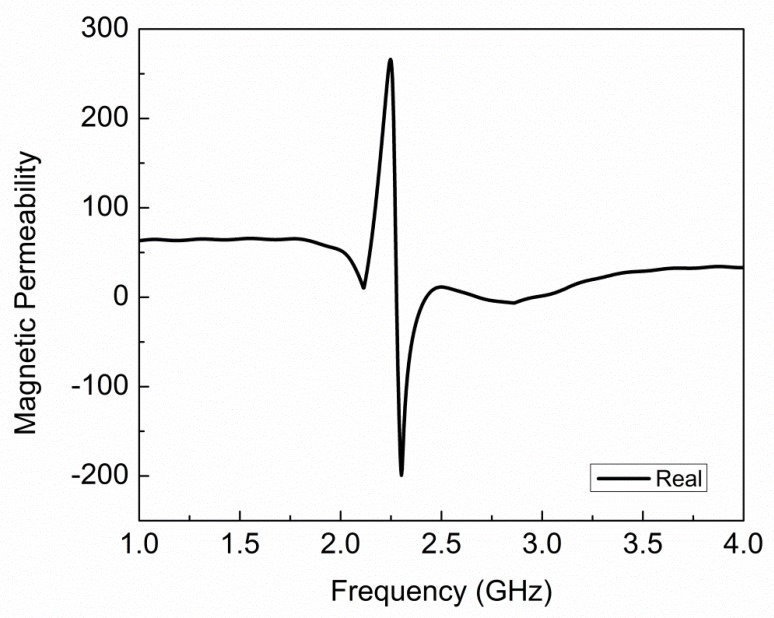
Extracted plot of magnetic permeability for proposed sensor, showing negative permeability from 2.27 GHz to 2.42 GHz, and maximum value of negative permeability is −199.2 at 2.30 GHz.

**Figure 6:**
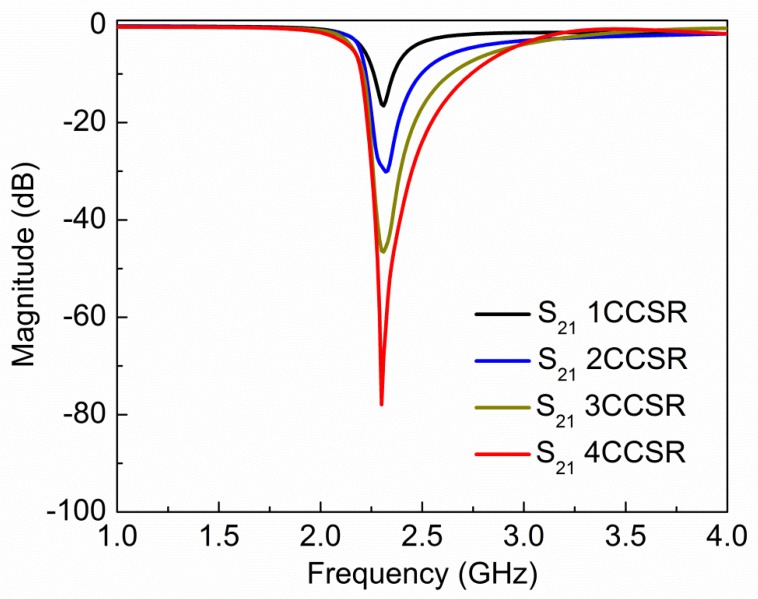
Simulated transmission coefficient of proposed sensor with one, two, three, and four complementary circular spiral resonator (CCSR) in the ground plane separated 1 mm apart. By increasing number of CCSR in the ground plane, notch depth and bandwidth increase but resonance frequency remains the same.

**Figure 7:**
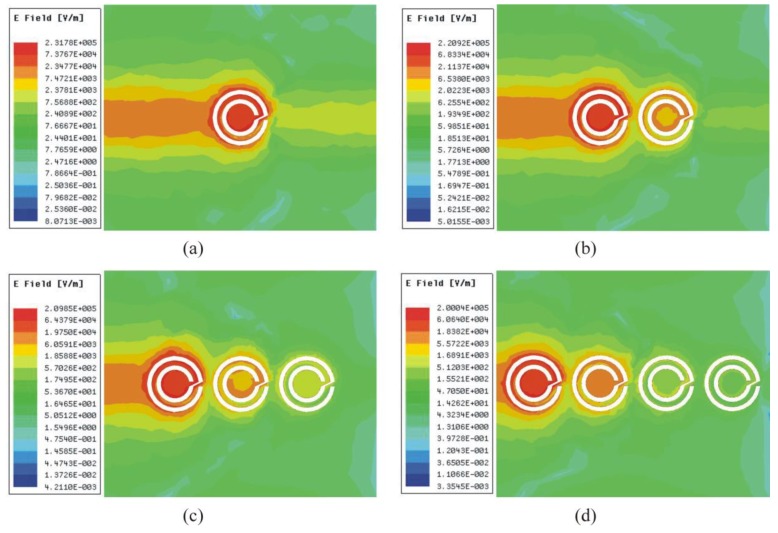
Distribution of electric field at resonance of proposed sensor based on (**a**) one CCSR, (**b**) two CCSR, (**c**) three CCSR, and (**d**) four CCSR.

The extracted plot of electric permittivity for the proposed sensor is plotted in Figure 4 and it shows negative permittivity from 2.10 GHz to 2.12 GHz, and the maximum value of negative permittivity is −3.57 at 2.11 GHz. The extracted plot of magnetic permeability for the proposed sensor is plotted in Figure 5 and it shows negative permeability from 2.27 GHz to 2.42 GHz, and the maximum value of negative permeability is −199.2 at 2.30 GHz. From Figure 4 and Figure 5, it is clear that the proposed sensor is giving negative values of permittivity and permeability, which is a property of metamaterials. The values of electric permittivity and magnetic permeability are dimensionless as they are relative to free space and can vary with the orientation of the sensor, temperature of environment, and molecular structure of the material. The number of CCSR structures in the ground plane of the proposed sensor is increased and their effect on resonance frequency, notch depth, and bandwidth are shown in Figure 6 and tabulated in Table 2. By increasing the number of CCSR in the ground plane, there is no effect on resonance frequency, but notch depth and bandwidth increase and the transmission curve becomes sharper. Since the resonance frequency of each CCSR structure is the same, there is no effect on resonance frequency, but each structure contributes to the notch depth. Due to the increase in notch depth, the bandwidth also increases. Figure 7 shows the electric field distribution at resonance frequency of the proposed sensor based on one, two, three, and four CCSR structures in the ground plane. The maximum magnitude of electric field is 2.31 × 10^5^ V/m for one CCSR in the ground plane and it is concentrated inside the inner ring. For two, three, and four CCSRs, the same electric field distributes among two structures and the maximum magnitude of electric field is 2.20 × 10^5^ V/m, 2.09 × 10^5^ V/m, and 2.0 × 10^5^ V/m, respectively. 

## 3. Sensitivity Analysis

**Figure 8:**
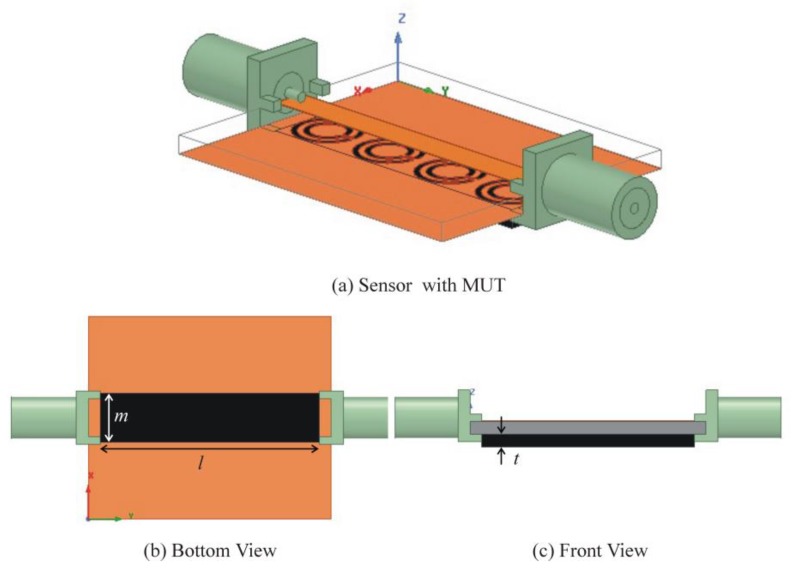
(**a**) Sensor based on four CCSR with materials under test (MUT) in the ground plane without air gap. (**b**) Bottom view of the sensor with MUT (*l* = 27 mm, and *m* = 6 mm). (**c**) Front view of the sensor with MUT (*t* = 1 mm).

**Figure 9:**
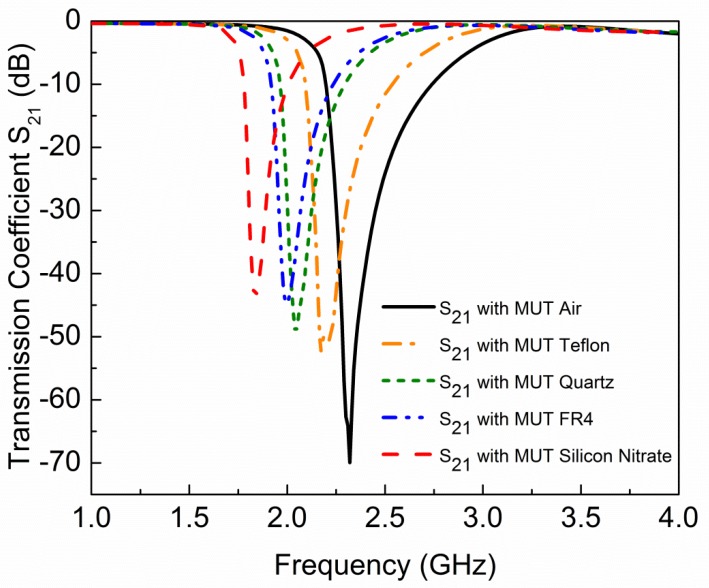
Simulated transmission coefficient *S*_21_ (dB) of sensor due to interaction with different MUTs. Resonance frequencies of sensors due to interaction with air, teflon, quartz, FR4, and silicon nitrate are 2.32 GHz, 2.17 GHz, 2.04 GHz, 2.0 GHz, and 1.84 GHz, respectively.

The basic operating principal of microwave sensors is to measure the change in resonance frequency due to interaction with the material under test (MUT). According to the theoretical development, the shift in resonance frequency of sensor due to interaction with the MUT can be expressed as [14]:

(9) $$\frac{\Delta f_{r}}{f_{r}}=\frac{\int_{\upsilon }(\Delta \varepsilon E_{1}\cdot E_{0}+\Delta \mu H_{1}\cdot H_{0})d\upsilon }{\int_{\upsilon }(\varepsilon_{0}{\left|E_{0}\right|}^{2}+\mu_{0}{\left|H_{0}\right|}^{2})d\upsilon }$$

where ∆*f_r_*, ∆*ε*, ∆*µ* are change in resonance frequency, permittivity, and permeability, respectively. *υ* is the perturbed volume. *E*_0_ and *E*_1_ are the electric fields distribution without and with perturbation, respectively. *H*_0_ and *H*_1_ are the magnetic fields distribution without and with perturbation, respectively. For sensitivity analysis, we selected the sensor based on four CCSR structures due to its sharp transmission curve. MUT is placed in the ground plane without air gap as shown in Figure 8. Sensitivity analysis is performed on permittivity perturbation of MUTs. Four MUTs (teflon, quartz, FR4, and silicon nitrate) with constant dimension (6 mm × 27 mm × 1 mm) are placed under the ground plane and their impact on transmission coefficient of the sensor is shown in Figure 9. Simulated resonance frequencies of the sensors due to interaction with air, teflon, quartz, FR4, and silicon nitrate are 2.32 GHz, 2.17 GHz, 2.04 GHz, 2.0 GHz, and 1.84 GHz, respectively. From Figure 9, it is clear that the relative permittivity of the MUT is inversely proportional to the resonance frequency of the sensor. Permittivity of MUT basically changes the total capacitance of the device, and resonance frequency due to interaction with MUT can be calculated as follows [15]:

(10) $$f=\frac{1}{2\pi \sqrt{L\left(C_{substrate}+C_{MUT}\right)}}$$

## 4. Fabrication and Measurement

**Figure 10:**
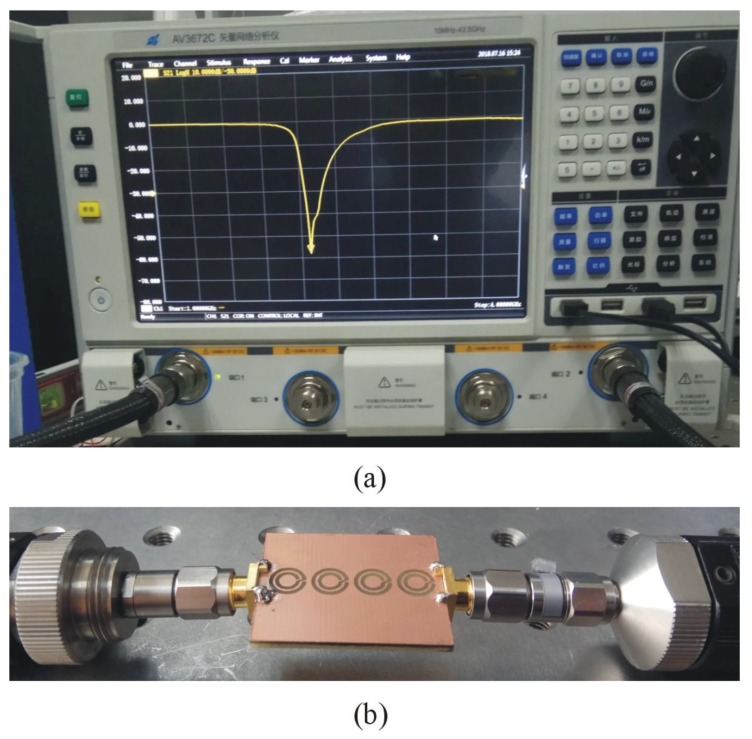
(**a**) Photograph of Vector Network Analyzer (VNA) for measurement of sensor. (**b**) Fabricated prototype of the sensor based on four CCSR.

**Figure 11:**
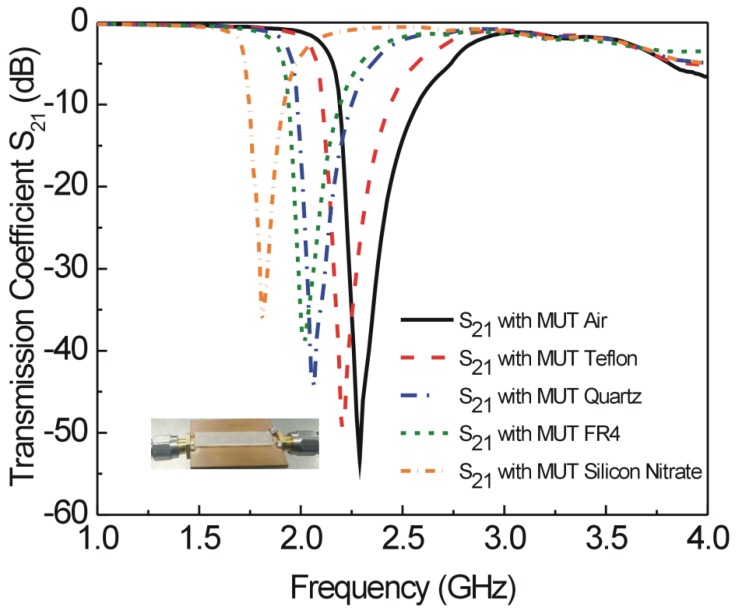
Measured transmission coefficient *S*_21_ (dB) of sensor due to interaction with different MUTs. Resonance frequencies of sensors due to interaction with air, teflon, quartz, FR4, and silicon nitrate are 2.29 GHz, 2.20 GHz, 2.06 GHz, 2.02 GHz, and 1.82 GHz, respectively.

The sensor based on four CCSR structures is fabricated on FR4 substrate using standard PCB fabrication technique and the fabricated prototype is shown in Figure 10b. Vector network analyzer (AV3672series) is used for measurement of transmission coefficient with a frequency sweep of 1 to 4 GHz as shown in Figure 10a. The fabricated sensor is used to measure the resonance frequencies of sensors due to interaction with air, teflon, quartz, FR4, and silicon nitrate as shown in Figure 11. Measured resonance frequencies of the sensors due to interaction with air, teflon, quartz, FR4, and silicon nitrate are 2.29 GHz, 2.20 GHz, 2.06 GHz, 2.02 GHz, and 1.82 GHz, respectively. For comparison, simulated and measured results for the sensor are tabulated in Table 3. The differences between simulated and measured results are very small and can be attributed to fabrication tolerance, conductor, dielectric and radiation losses.

## 5. Formulation

**Figure 12:**
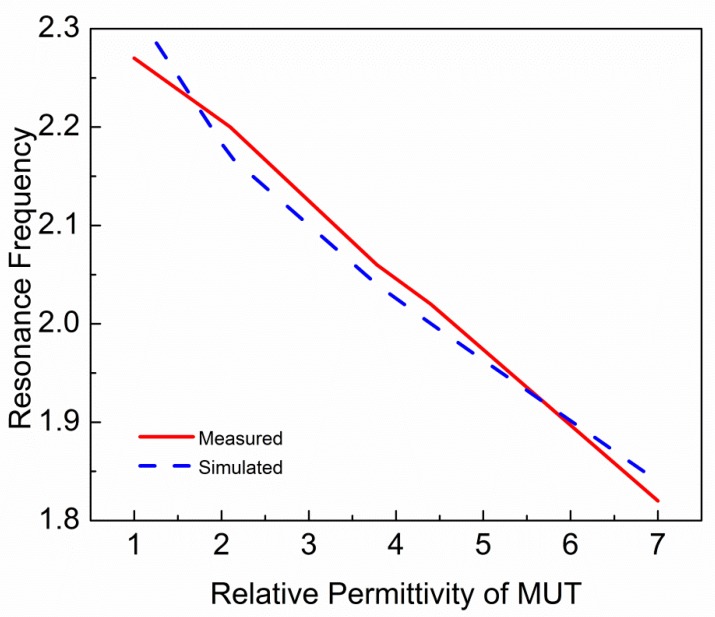
Relative permittivity of MUT versus resonance frequency of the sensor obtained by Electromagnetic (EM) simulation and VNA measurement. Relative permittivity of MUT is inversely proportional to the resonance frequency of sensor.

The transcendental equation for the proposed sensor is formulated with fitting parameters using measured results. As shown in Figure 11, the resonance frequency of the sensor varies with relative permittivity of MUT. This variation in resonance frequency can be expressed by the following equation [20]:

(11) $$f_{r,MUT}=f_{r,Air}\sqrt{\frac{\varepsilon_{eff,AIR}}{\varepsilon_{eff,MUT}}}$$

where *f_r,MUT_* and *f_r,AIR_* are resonance frequencies of the sensor with and without MUT, respectively. While *ε_eff,MUT_* and *ε_eff,AIR_* are effective permittivity of MUT and air, respectively. Figure 12 shows the relationship between relative permittivity of MUT and the resonance frequency of sensor due to interaction with MUT. This relationship shows that resonance frequency of the sensor is decreasing by increasing the relative permittivity of MUT. In Ref. [20], resonance frequencies are approximated with a parabolic equation with respect to relative permittivity of MUT. The parabolic equation is given as:

(12) $$f_{r,MUT}=A_{1}+A_{2}\varepsilon_{r}^{\prime }+A_{3}\varepsilon_{r}^{\prime }{}^{2}$$

where *ε_r_*’ is relative permittivity of MUT. *A*_1_, *A*_2_, and *A*_3_ are constant values of polynomial. The reference MUT is air, which has dielectric constant 1. For reference, MUT resonance frequency must be equal to *A*_1_, so Equation (12) can be expanded with respect to (*ε_r_*’ − 1),

(13) $$f_{r,MUT}=A_{1}+A_{2}\left(\varepsilon_{r}^{\prime }-1\right)+A_{3}{\left(\varepsilon_{r}^{\prime }-1\right)}^{2}$$

By measuring the results of standard materials (Air, Teflon, and FR4) for which dielectric constants are well known and curve fitting, the constant parameters of Equation (13) are extracted. Finally, the equation becomes:

(14) $$f_{r,MUT}=2.29-0.08297\left(\varepsilon_{r}^{\prime }-1\right)+0.00105{\left(\varepsilon_{r}^{\prime }-1\right)}^{2}$$

**Figure 13:**
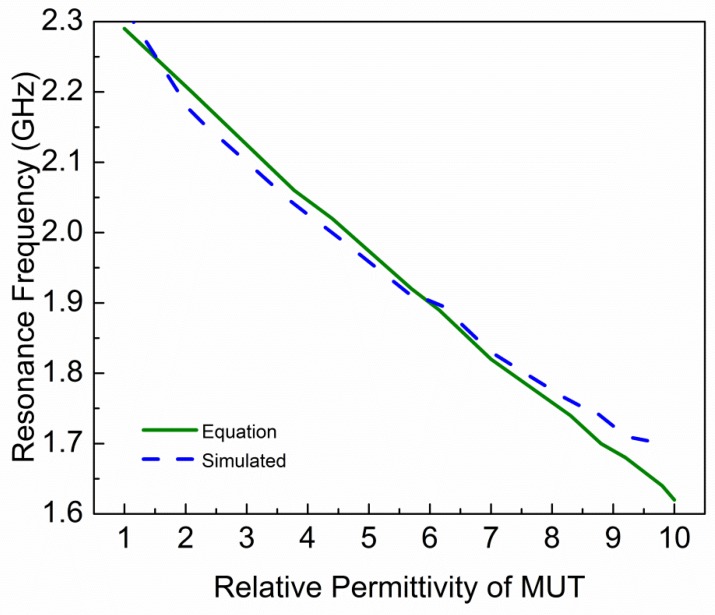
Relative permittivity of MUT versus resonance frequency of the sensor obtained by EM simulation and Equation (14).

Equation (14) can be used to predict resonance frequency of known MUTs with permittivity ranges from 1 to 10. To check validity of Equation (14), various MUTs are placed on the sensor and resonance frequencies are extracted through Electromagnetic (EM) simulation. In Figure 13, the solid line shows the resonance frequencies extracted from Equation (14) and dash line shows the resonance frequencies extracted through simulation. It is clear that Equation (14) is fairly reliable for the prediction of resonance frequency of the sensor due to the interaction with MUTs of permittivity ranges up to 10. To calculate the relative permittivity of unknown MUT, the transcendental equation can be expressed as:

(15) $$\varepsilon_{r}^{\prime }=\frac{0.08297-\sqrt{0.00688-0.0042\left(2.29-f_{r,MUT}\right)}}{0.0021}+1$$

Equation (15) can be used to calculate relative permittivity of unknown MUTs. In order to check the validity of Equation (15), measured resonance frequencies are used to calculate the relative permittivity of different MUTs and are tabulated in Table 4. It is clear that the relative permittivity values obtained from Equation (15) are very close to the actual values.

## 6. Conclusions

A complementary metamaterial sensor based on the microstrip transmission line and complementary circular spiral resonator (CCSR) was designed numerically and verified experimentally for nondestructive evaluation of dielectric substrates. Constitutive parameters for the proposed sensor were calculated using ANSYS electronics desktop. A sharp transmission curve was achieved using four CCSR structures in the ground plane. Sensitivity analysis was performed using permittivity perturbation of material under test (MUT). The shift in the resonance frequency of the sensor due to interaction with MUT is presented as a function of permittivity of MUT. The transcendental equation was derived for the sensor to predict the resonance frequency of dielectric materials. Simulated, measured, and formulated results are very close to each other within permittivity range from 1 to 10. The proposed design is very compact and fabrication is easy and inexpensive. Although the main emphasis of this work is for the evaluation of dielectric substrates, in the future the design will be improved for biosensing and security applications. 

## Figures and Tables

**Table 1:** ANSYS High Frequency Structure Simulator (HFSS) Simulation Condition.

Analysis Area	Size	25 × 30 × 50 mm^3^
	Boundary Condition	Radiation
Cells	Number	14,201
	Shape	Tetrahedron
Feed		Wave port (50 Ω)
Solution Type		Driven Model
Convergence condition determination		Maximum number of passes; 20 Maximum delta S; 0.02

**Table 2:** Effect of CCSR number on resonance frequency, notch depth, and bandwidth.

Number of CCSR in Ground Plane	Resonance Frequency (GHz)	Notch Depth (dB)	B.W at 3dB (GHz)
One	2.32	−16.54	0.36
Two	2.32	−30.10	0.88
Three	2.32	−46.54	1.01
Four	2.32	−77.93	0.96

**Table 3:** Simulated and measured results of sensor for different MUTs.

Material Under Test (MUT)	Relative Permittivity of MUT	Simulated Resonance Frequency (GHz)	Measured Resonance Frequency (GHz)	Difference Between Simulation and Measurement
Air	1	2.32	2.29	0.05
Teflon	2.1	2.17	2.20	0.03
Quartz Glass	3.78	2.04	2.06	0.02
FR4 Epoxy	4.4	2.0	2.02	0.02
Silicon Nitrate	7	1.84	1.82	0.02

**Table 4:** Relative permittivity evaluation using measured result.

Material Under Test (MUT)	Relative Permittivity (*ε_r_*’)	Equation (15) Calculation or *ε_r_*’
Air	1	1.01
Teflon	2.1	2.11
Quartz Glass	3.78	3.88
FR4 Epoxy	4.4	4.41
Silicon Nitrate	7	7.15

## References

[1] Tiwari N.K., Singh S.P., Akhtar M.J. (2019). Novel Improved sensitivity planar microwave probe for adulteration detection in edible oils. IEEE Microw. Wirel. Components Lett..

[2] Kumari R., Patel P.N., Yadav R. (2018). An ENG-inspired microwave sensor and functional technique for label-free detection of aspergillus Niger. IEEE Sens. J..

[3] Trabelsi S., Nelson S.O. (2016). Microwave sensing of quality attributes of agricultural and food products. IEEE Instrum. Meas. Mag..

[4] Abdolrazzaghi M., Khan S., Daneshmand M. (2018). A Dual-Mode Split-Ring Resonator to Eliminate Relative Humidity Impact. IEEE Microw. Wirel. Components Lett..

[5] Chuma E.L., Iano Y., Fontgalland G., Bravo Roger L.L. (2018). Microwave sensor for liquid dielectric characterization based on metamaterial complementary split ring resonator. IEEE Sens. J..

[6] Falcone F., Lopetegi T., Baena J. (2004). Effective negative epsilon stopband microstrip lines based on complementary split ring resonators. IEEE Microw. Wirel. Components Lett..

[7] Pendry J.B., Holden A.J., Robbins D.J., Stewart W.J. (1999). Magnetism from conductors and enhanced nonlinear phenomena. IEEE Microw. Wirel. Components Lett..

[8] Baena J.D., Bonache J., Martín F., Sillero R.M., Falcone F., Lopetegi T., Laso M.A.G., García-García J., Gil I., Portillo M.F. (2005). Equivalent-circuit models for split-ring resonators and complementary split-ring resonators coupled to planar transmission lines. IEEE Trans. Microw. Theory Tech..

[9] Zarifi M.H., Deif S., Abdolrazzaghi M., Chen B., Ramsawak D., Amyotte M., Vahabisani N., Hashisho Z., Chen W., Daneshmand M. (2017). A microwave ring resonator sensor for early detection of breaches in pipeline coatings. IEEE Trans. Ind. Electron..

[10] Mohd Bahar A.A., Zakaria Z., Ab Rashid S.R., Isa A.A.M., Alahnomi R.A. (2017). High-efficiency microwave planar resonator sensor based on bridge split ring topology. IEEE Microw. Wirel. Components Lett..

[11] Puentes M., Maasch M., Schubler M., Jakoby R. (2012). Frequency multiplexed 2-dimensional sensor array based on split-ring resonators for organic tissue analysis. IEEE Trans. Microw. Theory Tech..

[12] Boybay M.S., Ramahi O.M. (2012). Material characterization using complementary split-ring resonators. IEEE Trans. Instrum. Meas..

[13] Haq T.U., Ruan C., Ullah S., Kosar A. Reconfigurable Ultra Wide Band Notch Filter based on Complementary Metamaterial. Proceedings of the 2018 IEEE Asia-Pacific Conference on Antennas and Propagation (APCAP).

[14] Lee C.S., Yang C.L. (2014). Thickness and permittivity measurement in multi-layered dielectric structures using complementary split-ring resonators. IEEE Sens. J..

[15] Lee C.S., Yang C.L. (2014). Complementary split-ring resonators for measuring dielectric constants and loss tangents. IEEE Microw. Wirel. Components Lett..

[16] Albishi A.M., Ramahi O.M. (2018). Highly sensitive microwaves sensors for fluid concentration measurements. IEEE Microw. Wirel. Components Lett..

[17] Zhang X., Ruan C., Haq T., Chen K. (2019). High-Sensitivity Microwave Sensor for Liquid Characterization Using a Complementary Circular Spiral Resonator. Sensors.

[18] HONG J.-S. (2011). Microstrip filters for RF/Microwave Applications.

[19] Chen X., Grzegorczyk T.M., Wu B.I., Pacheco J., Kong J.A. (2004). Robust method to retrieve the constitutive effective parameters of metamaterials. Phys. Rev. E Stat. Physics Plasmas Fluids Relat. Interdiscip. Top..

[20] Lim S., Kim C.Y., Hong S. (2018). Simultaneous Measurement of Thickness and Permittivity by Means of the Resonant Frequency Fitting of a Microstrip Line Ring Resonator. IEEE Microw. Wirel. Components Lett..

